# Genetic polymorphisms of the RAS-cytokine pathway and chronic kidney disease

**DOI:** 10.1007/s00467-008-0816-z

**Published:** 2008-07-01

**Authors:** Craig Wong, Peter Kanetsky, Dominic Raj

**Affiliations:** 1 Department of Pediatrics, Division of Nephrology, Albuquerque, NM USA; 2 Department of Biostatistics & Epidemiology, University of Pennsylvania, Philadelphia, PA USA; 3 Division of Nephrology, Medicine University of New Mexico Health Sciences Center, Albuquerque, NM USA; 4 Department of Pediatrics, Division of Nephrology, MSC10-5590, 1 University of New Mexico, Albuquerque, NM 87131-0001 USA

**Keywords:** Chronic kidney disease, Cardiovascular disease, Genetic, Cytokine, Polymorphism, Progression, Pediatric

## Abstract

Chronic kidney disease (CKD) in children is irreversible. It is associated with renal failure progression and atherosclerotic cardiovascular (CV) abnormalities. Nearly 60% of children with CKD are affected since birth with congenital or inherited kidney disorders. Preliminary evidence primarily from adult CKD studies indicates common genetic risk factors for CKD and atherosclerotic CV disease. Although multiple physiologic pathways share common genes for CKD and CV disease, substantial evidence supports our attention to the renin angiotensin system (RAS) and the interlinked inflammatory cascade because they modulate the progressions of renal and CV disease. Gene polymorphisms in the RAS-cytokine pathway, through altered gene expression of inflammatory cytokines, are potential factors that modulate the rate of CKD progression and CV abnormalities in patients with CKD. For studying such hypotheses, the cooperative efforts among scientific groups and the availability of robust and affordable technologies to genotype thousands of single nucleotide polymorphisms (SNPs) across the genome make genome-wide association studies an attractive paradigm for studying polygenic diseases such as CKD. Although attractive, such studies should be interpreted carefully, with a fundamental understanding of their potential weaknesses. Nevertheless, whole-genome association studies for diabetic nephropathy and future studies pertaining to other types of CKD will offer further insight for the development of targeted interventions to treat CKD and associated atherosclerotic CV abnormalities in the pediatric CKD population.

## Introduction

Chronic kidney disease (CKD) is irreversible and progressive [1]. In children, CKD is underappreciated, understudied, and an important cause of morbidity and mortality [1, 2]. Furthermore, adjusted mortality rates since 1991 among the pediatric end-stage renal disease (ESRD) population increased by 5% to 26.6 per million general population in 2005; and cardiovascular (CV) mortality among pediatric ESRD patients has increased from 17.7 deaths per 1,000 patient years at risk in 1991 to 23.4 in 2005 [3]. Children with CKD live with the consequences of abnormal renal function for their entire lives, with nearly 60% affected since birth with congenital or inherited kidney disorders [2]. Diabetic nephropathy and hypertension, which are the dominant causes of CKD in adults, are rare causes of CKD in childhood. CKD in children is the result of heterogeneous diseases of the kidney and urinary tract that range from common congenital malformations of the urinary tract to rare inborn errors of metabolism. Although some patients have stable kidney function for years, others have a rapid decline in function. The factors associated with an accelerated decline in kidney function include: the cause of CKD, proteinuria, hypertension, anemia, hyperphosphatemia, and metabolic acidosis [4–9]. In common with children and adults with CKD, progression to kidney failure occurs via a final common pathway characterized by progressive interstitial fibrosis, peritubular capillary loss with hypoxia, and destruction of functioning nephrons because of tubular atrophy [10]. Despite the diverse initiating and secondary factors noted above, CKD progression is strongly influenced by common inflammatory mechanisms [11].

CKD is a well-known risk factor for atherosclerotic CV disease [12–16]. Children with kidney failure receiving chronic dialysis have a cardiac death rate 1,000-fold higher compared with children in the general population [15]. Children with mild to moderate CKD have a high prevalence of traditional risk factors for atherosclerotic CV disease, including hypertension, hyperlipidemia, and elevated homocysteine levels [17–19]. Left ventricular hypertrophy (LVH), a pathophysiologic adaptation of the myocardium, is viewed as a marker for early CV disease in pediatric patients with CKD [20, 21]. The long-standing and progressive atherosclerotic CV abnormalities that begin in childhood CKD contribute to the increased CV morbidity in adulthood [22]. CKD promotes maladaptive interactions between the heart and kidneys, which in turn amplifies the progressive failure of these organs [23]. The emerging evidence suggests that cytokines may play a vital regulatory role in initiation and progression of both renal and CV disease in patients with CKD [24].

Preliminary evidence primarily from adult CKD studies [24] indicates common genetic risk factors for CKD and atherosclerotic CV disease. Although multiple physiologic pathways share common genes for CKD and CV disease, substantial evidence [10, 25–28] supports our attention to the renin angiotensin system (RAS) and the interlinked inflammatory cascade because they modulate the progression of renal and CV disease. Current research suggests that the natural variations of the genes involving the RAS-cytokine pathway influence the rate of progressions for renal and CV disease in CKD patients [24]. Insights gained by understanding how variations in this pathway influence the progressions of renal and CV disease will lead to hypotheses for targeted interventions to treat CKD and associated atherosclerotic CV abnormalities in the pediatric CKD population. This article reviews clinically relevant candidate genes of the RAS-cytokine pathway and the fundamentals of genotype–phenotype association studies.

## CKD–CV disease link: RAS-cytokine pathway

Intervention trials in adults with CKD have demonstrated that blockade of the RAS slow progression of renal disease via antihypertensive and anti-inflammatory mechanisms [26–28]. The RAS generates circulating angiotensin II (AT2), which regulates blood pressure and intravascular volume. In contrast to its endocrine function, tissue RAS produces AT2 that is involved in autocrine and paracrine signaling within all bodily organs, including the heart, blood vessels, and kidneys [29]. Tissue RAS exerts a pivotal role in the regulation of cytokine signaling, potentially modulating the inflammatory response associated with renal disease progression and susceptibility for CV dysfunction.

**Fig. 1:**
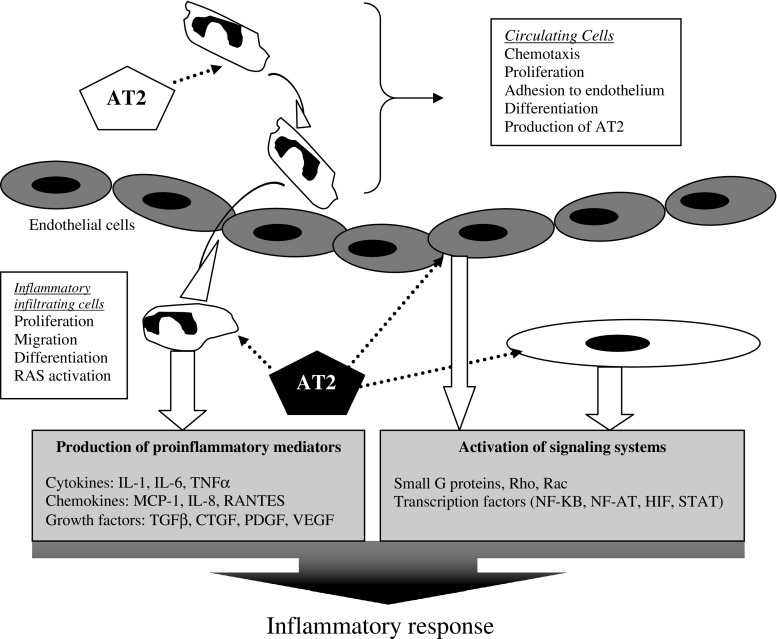
Activation of the renin angiotensin system (RAS) and an increase in the local production of angiotensin II (AT2) triggers the inflammatory host response

Tissue RAS via AT2 regulates the cytokine pathway responsible for progressive injury in the kidney and heart [25, 30–32]. As depicted in Fig. 1, activation of tissue RAS increases the local production of AT2. After AT2 stimulates the AT2 receptor, a number of signaling systems are triggered, including that of nuclear factor kappa B (NF-κB), which is responsible for upregulation of proinflammatory cytokines [33]. The cytokine signaling modulates endothelial dysfunction, adhesion and migration of circulating immune cells (monocyte, leukocytes, or neutrophils) into the interstitium, and activation of resident fibroblasts [10, 11]. Cytokines are soluble polypeptides that act as important humoral modulators in immunoregulation, hematopoiesis, and inflammation. Cytokines act in a highly complex coordinated network with considerable overlap and redundancy between the function of individual cytokines. Being pleiotropic in their actions, these molecules can induce or repress their own synthesis as well as that of other cytokines and cytokine receptors [24, 34].

In the kidney, the inflammatory host response leads to renal interstitial fibrosis and progression [10]. These actions within the kidney are mediated by proinflammatory [tumor necrosis factor (TNF)-α, interleukin (IL)-1, IL-6] and profibrotic cytokines [TGF-β and plasminogen activator inhibitor (PAI)-1] [10, 35]. Proteinuria stimulates interstitial inflammation and fibrosis in the kidney; it also is a risk factor for future decline in kidney function [36]. NF-κB activity is stimulated by albumin [37] and is the pathway that links proteinuria and tubulointerstitial inflammation and fibrosis in the kidney [38, 39]. In the heart, the AT2-stimulated inflammatory response leads to LVH [40, 41]. The myocardial hypertrophy is caused by an increase in cell size and accompanied by changes in gene expression in response to AT2 [42, 43]. In addition to AT2, proinflammatory cytokines IL-1, IL-6, TNF-α, and TGF-β are responsible for myocyte hypertrophy and interstitial fibrosis [44, 45]. In the blood vessel, the AT2 pathway is the molecular mechanism leading to atherosclerosis [25, 32]. There is preliminary evidence indicating impaired flow-mediated dilation among hypertensive patients with mutations in the promoter region of the NF-κB gene [46]. Hence the upregulation of RAS-cytokine pathway activity is associated with renal progression, LVH, and atherosclerosis. The magnitude of this response may depend on genetic polymorphisms, which may either increase or decrease expression of these genes [24, 47].

## Genetic polymorphisms

Different versions of a gene at a specific chromosomal location, or loci, that encode a trait are called alleles (see Table 1 for a glossary of common terms). Complementary alleles are inherited from each parent. A change in one nucleotide (base pair) within a gene is called a single nucleotide polymorphism (SNP). About 11 million SNPs with minor allele frequencies (MAF) of at least 1% are estimated to exist in the human genome. SNPs that affect native protein function, i.e. functional SNPs, can occur in gene promoter regions, coding regions, splice junctions, and 3’-untranslated regions (UTR) and may be causally involved in the etiology of human disease. Other types of polymorphisms include insertion/deletion (indel) polymorphisms and mini- and microsatellites (di-, tri-, and tetranucleotide repeats) [48]. Variation in phenotypic expression of a gene may be affected by epigenetic factors, where gene expression is affected by mechanisms other than alterations in the nucleotide sequence, but this is beyond the scope of this review.

**Table 1:** Glossary of common genetic terms

**Alleles:** Alternate sequences of the same gene, one inherited from each parent.
**Biological pathway:** A set of proteins that interact to produce normal and abnormal physiology.
**Candidate gene:** A gene in which variants could plausibly explain a given phenotype, such as severity of disease or variable response to drug. Methods to identify candidate genes include basic science studies, identifying DNA sequences conserved across species, human genetics, epidemiologic association studies, or genome-wide analyses.
**Epigenetics:** Heritable change in the pattern of gene expression mediated by mechanism other than alterations in the primary nucleotide sequence of gene.
**Genome:** The collection of all DNA in an organism. Only a small proportion (probably <3%) of human genomes encodes proteins.
**Genotype:** The genetic makeup of an individual, which may refer to the whole genome or to specific genes or regions of genes.
**Haplotype:** A set of genetic variants that are inherited together. Polymorphisms that are coinherited more often than by chance alone are in linkage disequilibrium (LD). Haplotype blocks may include many individual polymorphisms in high LD; as a result, establishing genotype at any single polymorphic site with such a block may establish genotypes at linked sites within the block. Individual single-nucleotide polymorphisms (SNPs) that can be used to establish genotype within a haplotype block are termed tag SNPs.
**Heterozygous:** Having different alleles in a specific region of DNA.
**Homozygous:** Having the same alleles in a specific region of DNA
**Phenotype:** Measurable characteristics of an organism. These may derive from genotype, environment, or the combination. Organisms with the same phenotype can have different genotypes.
**Polymorphisms:** DNA variants that are common, often defined as >1% in a given population. Polymorphisms can be in coding regions (where they may be synonymous or nonsynonymous) or, more commonly, in noncoding regions, and often vary by ethnicity. The most common type of polymorphism is a change in one nucleotide (base pair) in a DNA sequence, referred to as an SNP. Other polymorphisms are insertion and deletion of multiple sequential nucleotides (indels); variable numbers of repeats, such as doublets or triplets; or large-scale duplications or deletions. Although some genetic variants are known to alter protein abundance or function, the functional consequences of most polymorphisms are unknown.
**Tag SNPs:** These are maximally informative SNPs that characterize common haplotypes.

The study of individual SNPs has yielded exciting insight into the factors involved in CKD progression. Although candidate-gene-based approaches are a logical first step, they are unlikely to provide a complete answer. The progressions to renal and CV disease are complex traits involving multiple genes. As discussed later, the cooperative efforts among scientific groups and the availability of robust and affordable technologies that can identify thousands of SNPs across the genome make genome-wide association studies an attractive paradigm for studying polygenic diseases such as CKD. Whole-genome association studies are being used to identify the genetic basis for CKD, with large consortiums investigating the genetic predisposition to diabetic nephropathy in Europe and North America [49, 50].

Although a complete overview of all known genetic polymorphisms of the RAS-cytokine pathway is beyond the scope of this review, a complete listing can be found at the National Center for Biotechnology Information (NCBI) SNP database (dbSNP) (https://doi.org/www.ncbi.nlm.nih.gove/projects/SNP/). There are polymorphisms of the RAS-cytokine genes that have been reported to be associated with renal progression and/or CV morbidity and are summarized below (Table 2). Despite some of the potential weaknesses of the studies included, these candidate-gene association studies offer some preliminary information worthy of further investigation**.**

**Table 2:** Candidate-gene polymorphisms and associations with renal and cardiovascular (CV) diseases among subjects with chronic kidney disease (CKD)

Author	Gene and genotype^a^	Study population/study type^b^	Sample size (*n*)	Clinical significance
**Renin Angiotensin System (RAS)**				
Boright A [53]	ACE haplotypes	US diabetic nephropathy CKD (DCCT-EDIC)/cohort study	Restricted to Caucasian cohort = 1,365	The genetic variation of the ACE gene is associated with microalbuminuria and diabetic nephropathy.
Hadjadj S [49]	ACE insertion/deletion polymorphism	European diabetic nephropathy CKD/case-control study	Adult diabetic nephropathy cases = 1,057, controls = 1,127	The haplotype including the ACE deletion allele was associated with diabetic nephropathy.
Haszon I [57]	ACE insertion/deletion polymorphism	European Vesicoureteral reflux CKD/case-control study	Pediatric VUR cases = 77, controls = 80	A deletion at both alleles is linked to renal scarring in VUR.
Hohenfellner K [55]	ACE insertion/deletion polymorphism	European CKD (Nutritional Treatment of Chronic Renal Failure in Childhood Study)/cohort study	Pediatric cohort = 95	A deletion at both alleles is linked to a higher risk of renal progression among children with congenital renal malformation.
Lovati E [58]	ACE insertion/deletion polymorphism	European ESRD/case-control study	Adult ESRD cases = 260, controls = 327	A deletion at both alleles is linked to a higher risk of renal progression among adults.
Ng D [54]	ACE insertion/deletion haplotype	US diabetic nephropathy CKD/case-control study	Adult Caucasians type 2 with diabetic nephropathy cases = 291, controls = 167	The deletion allele haplotype is associated with diabetic nephropathy.
Papp F [56]	ACE insertion/deletion polymorphism	European ESRD/case-control study	Pediatric ESRD cases = 20, controls = 150	A deletion at both alleles is linked to ESRD.
Hsu C [64]	AGT-6 G>A	US CKD (ARIC Study)/ cohort study	Restricted to Adult African American CKD cohort = 3,381 ^c^	Genotype A/A is linked to a higher risk of renal progression among African American CKD patients.
Reis K [111]	AGT M235T	European kidney transplant/case-control study	Adult kidney transplant cases = 82, controls = 100	Genotype Thr/Thr linked to chronic allograft dysfunction.
Buraczynska M [66]	AT1R A>C	European ESRD/case-control study	Adult ESRD cases = 745, controls = 520	Genotype C/C or A/C is linked to higher risk of renal progression in adults.
**Interleukin (IL)-1**				
Wetmore JB [73]	IL-1α g.-889 C>T	US ESRD/case-control study	Adult ESRD cases = 239, controls = 252	Genotype T/T is linked with risk for ESRD.
Amoli M [82]	IL-1β g.-511 C>T	European Henoch-Schonlein purpura disease (HSP)/case-control study	Adult and pediatric HSP cases = 49, controls = 146	Carriage of the T allele linked to severity of renal involvement with Henoch-Schonlein purpura.
**Interleukin-1 Receptor Antagonist**				
Buraczynska M [83]	IL-2RN*2	European ESRD/case-control study	Adult ESRD cases = 602, controls = 433	Homozygous for the IL2RN*2 allele linked with more rapid progression in patients with glomerulonephritis and diabetic nephropathy and risk for ESRD.
Wetmore JB [73]	IL-1RN*2	US ESRD/case-control study	Adult ESRD cases = 239, controls = 252	Homozygous for the IL2RN*2 allele is linked to risk for ESRD.
Watanabe M [81]	IL-12N*2	Japanese IgA nephropathy CKD /case-control study	Adult IgA Nephropathy cases = 106, controls = 74	Carriage of the IL2RN*2 allele is linked to severe proteinuria and increased creatinine in IgA nephropathy.
**Interleukin-6**				
Balakrishnan V [72]	g.-174G>C	US ESRD (HEMO Study)/cross-sectional study	Adult ESRD cohort = 187	Genotype G/G or G/C is linked to increased comorbid conditions and decreased functional status among dialysis patients.
Losito A [86]	g.-174G>C	European ESRD/case-control study	Adult ESRD cases = 161, controls = 169	Carriage of the C allele linked to LVH in hemodialysis patients, especially those with diabetes.
Muller-Steinhardt M [87]	g.-174G>C	European Kidney transplant/cohort study	Adult kidney transplant cohort = 158	Carriage of the C allele linked to decreased kidney allograft survival.
**Interleukin-10**				
Girndt M [88]	g.-1082 G>A	European ESRD/cohort study	Adult ESRD cohort = 300	The A/A genotype is linked to a lower production of IL-10 and increased CV morbidity
**Tumor Necrosis Factor (TNF)-α**				
Balakrishnan V [72]	g.-308 G>A	US ESRD (HEMO Study)/cross-sectional study	Adult ESRD cohort = 187	Genotype A/A or A/G is linked to low serum albumin, increased comorbid conditions, and decreased functional status among dialysis patients.
**Transforming Growth Factor-β**				
Sato F [106]	g.-509C>T and g.+869T>C	Japanese IgA Nephropathy CKD/cross-sectional study	Adult IgA nephropathy cases = 329, controls = 297	The -509C/C and 869C/C genotypes are linked with heavy proteinuria and mesangial cell proliferation in patients with IgA nephropathy.
Rao M [107]	g.+915G>C	US ESRD (HEMO Study)/cross-sectional, and cohort studies	Adult ESRD cohort = 187	Genotype G/C vs G/G was linked with risk for prevalent vascular disease, new onset cardiac morbidity and cardiac mortality in HD patients.
**Plasminogen Activator Inhibitor (PAI)-1**				
Aucella F [113]	4G/5G	European ESRD/cohort study	Adult ESRD cohort = 417	Genotype 4G/4G is linked to increased risk for fatal MI among HD patients.
Reis K [111]	4G/5G	European Kidney transplant/case-control study	Adult kidney transplant cases = 82, controls = 100	Carriage of the 4G allele linked to chronic allograft dysfunction.
Wong A [112]	4G/5G	Chinese Systemic lupus erythematosus CKD/case-control study	Adult diabetic nephropathy cases = 95, controls = 46	Genotype of the 4G/4G linked to increased severity lupus nephritis among SLE patients.

*ACE* angiotensin converting enzyme,* AGT* angiotensin,* AT1R* angiotensin II type 1 receptor,* DCCT* Diabetes Control and Complications Trial,* EDIC* Epidemiology of Diabetes Interventions and Complications,* ESRD* end-stage renal disease,* ARIC* Arthrosclerosis Risk in Communities,* HEMO* hemodialysis,* VUR* vesicoureteral reflux,* HD* hemodyalysis, * SLE* systemic lupus erythematosus, *MI* myocardial infarction

^a^ The gene and gene polymorphism of interest

^b^ Study population is given to indicate potential for population stratification and type of CKD population (name of cohort study)/study type by: cross-sectional, cohort, or case control

^c^ In the study by Hsu et al., 3,449 subjects had AGT genotyping, whereas 3,381 subjects had both AGT and AT1R genotyping

### The renin-angiotensin system

Polymorphisms in the RAS system are associated with clinically significant renal and CV disease morbidity and thought to occur through a proinflammatory mechanism. The inflammatory response is activated by the RAS through the recruitment of proinflammatory cells to the site of injury [51, 52] and the upregulation of adhesion molecules on vascular endothelial cells and smooth muscle cells [30].

A naturally occurring variant in the angiotensin-converting enzyme (ACE) gene, located on 17q23, is a 250-base pair deletion in intron 16. The D/D genotype is associated with a high ACE level, whereas the I/I genotype is associated with a low ACE level. In subjects with type 1 and type 2 diabetes, the haplotype insertion allele of the ACE gene has been associated with lower risk of diabetic nephropathy compared with the haplotypes including the deletion allele [49, 53, 54]. The D/D genotype has been associated with renal progression in children and adults with CKD [55–58]. The D/D polymorphism has been associated with LVH and QTc interval prolongation in patients with ESRD [59, 60].

Polymorphisms in angiotensinogen (AGT), located on 1q42-q43, are associated with increase in risk for renal progression and CV disease. Reported frequently in the literature, the M235T SNP is a methionine (Met) to threonine (Thr) amino acid substitution at codon 235. The Thr/Thr genotype is associated with an increased risk for hypertension in the general population [61, 62] and in kidney transplant patients with chronic allograft dysfunction. Investigators have recently recommended a name change of the SNP M268T for the substitution at amino acid 268, to be consistent with accepted human gene mutation nomenclature [63]. Another SNP is the AGT-6 G/A promoter variant, which is associated with a higher risk for renal progression in the African American CKD population [64].

A polymorphism in the AT2 type 1 receptor (AT1R) gene polymorphism, located on chromosome 3 (3q21-q25) [65], has also been associated with risk of renal progression and CV disease. The polymorphism of interest is a nucleotide change from an adenine (A) to cytosine (C) in the 3′-UTR at nucleotide 1166. The C/C genotype has been associated with a more rapid onset of renal failure compared with those with the A/A genotype [66]. Furthermore, the C/C genotype also is associated with the development of hypertension and coronary artery disease [67, 68].

### Interleukin-1 and IL-1 receptor antagonist (IL-1Ra)

The IL-1 family consists of two proinflammatory cytokines, IL-1α and IL-1β, and a naturally occurring anti-inflammatory agent, the IL-1Ra. The balance between IL-1 and IL-1Ra in local tissues plays an important role in the susceptibility to and severity of many diseases [69, 70]. Plasma IL-1 and IL-1Ra have been shown to predict cardiovascular outcome [71] and mortality in ESRD [72]. The genes of the IL-1 complex map to the 430-kb region on the long arm of chromosome 2. The IL-1α gene has an SNP g.-889C>T, which is a base pair change from a cytosine to a thymine (C→T) [73]. The IL-1β gene has an SNP g.-511C>T, which has a base pair change exchange (C→T) [73] and g.+3953 [74]. The IL-1Ra gene contains a variable number of tandem repeat (VNTR) polymorphisms in intron 2 (IL-1RN) [75]. The IL-1RN allele 2 is related to increased production of IL-1β [76]. Polymorphisms in IL-1α have been associated with ESRD [73]. Gene polymorphisms of IL-1β and IL-1RN have been associated with hypertension [77], atherosclerosis [78–80], CAD [78–80], and progression of renal disease [81–83].

### Interleukin-6 (IL-6)

IL-6 is a proinflammatory cytokine that stimulates the production of C-reactive protein (CRP) and fibrinogen. There is a promoter polymorphism at position –174 of the IL-6 gene (g.-174G>C). Carriage of the C allele is associated with higher levels of IL-6 production in response to pathologic stimuli [84]. The polymorphism is associated with a higher risk of CV disease in the general population [84, 85]. In dialysis patients, the carriage of the C allele is associated with high blood pressure, LVH, and decrease in functional status [47, 86]; in kidney transplant recipients, it is associated with decreased graft survival [87].

### Interleukin-10 (IL-10)

IL-10 attenuates the inflammatory response [47]. Decreased production of IL-10 is associated with increased CRP and higher cardiovascular mortality [88]. The IL-10 gene is located on 1q31-32 and is composed of five exons and has SNPs at positions g.-592 C>A, g.-819 C>T, and g.-1082 G>A [89, 90]. The low producer genotype A/A of the g.-1082G>A SNP is associated with increased CV mortality in ESRD patients [88].

### Tumor necrosis factor-α (TNF-α)

TNF-α production is stimulated by AT2 and associated with tubulointerstitial fibrosis [91]. Furthermore, elevated TNF-α levels are associated with CV disease comorbidities: coronary artery disease [92], LVH [93], and congestive heart failure [94]. The TNF-α gene is located on chromosome 6 and is highly polymorphic. Numerous promoter-region SNPs exist and are located at the upstream positions -1031, -863, -857, -851, -419, -376, -308, -238, -163, and -49 relative to the transcription start site; another SNP is at +488 in the intron [95, 96]. In hemodialysis patients, the polymorphism g.-308 G>A in the promoter region of the TNF-α gene has been associated with significant comorbidity; the carriage of the A allele is associated with a low serum albumin, higher burden of comorbid conditions, and a low Karnofsky score [47].

### Transforming growth factor-β (TGF-β)

TGF-β is a cytokine that regulates cell growth, differentiation, and extracellular matrix production [97]. TGF-β transmits the profibrotic signaling of AT2 that promotes interstitial fibrosis in the kidney [98]. Blockade of AT2 by ACE inhibitors and AT2 receptor blocker (ARB) drugs reduces intrarenal TGF-β [99]. Furthermore, TGF-β is responsible for the production of additional fibrosis-promoting molecules such as connective tissue growth factor (CTGF) and PAI-1 [10]. Overproduction of TGF-β1 is associated with renal progression [10, 100], hypertension [101], and LVH [102]. Several polymorphisms have been identified in the TGF-β gene [103]. There are two polymorphisms in the signal peptide sequence Leu10->Pro (g.+869T>C), Arg25→Pro (g.+915G>C) associated with higher production of TGF-β1 [104, 105]. Gene polymorphisms in TGF-β1 have been associated with an increase in proteinuria and mesangial cell hypertrophy in patient with IgA nephropathy [106]. Furthermore, the +915G/C genotype at codon 25 (Arg/Pro) may be a genetic susceptibility factor for the development of atherosclerosis due to the genotype’s association with an increased risk for cardiac morbidity and cardiac-specific mortality in hemodialysis (HD) patients [107].

### Plasminogen activator inhibitor-1 (PAI-1)

Upregulation of PAI-1 favors extracellular matrix accumulation and fibrosis by inhibiting fibrinolysis [108]. AT2 signaling via the type 1 receptor increases the production of PAI-1 [108]. Furthermore, regression of sclerosis is associated with blockade AT2 and a reduction in PAI-1 [109]. The gene polymorphism of interest is a 4G/5G insertion/deletion 675 base pairs from the start of the promoter. The polymorphism affects the binding of nuclear proteins involved in the regulation of PAI-1 gene transcription, leading to higher rate of synthesis with the 4G/4G genotype [110]. The genotype is associated with chronic kidney allograft nephropathy [111] and increased activity of lupus nephritis [112], The PAI-1 4G/5G polymorphism is associated with fatal and nonfatal myocardial infarction in dialysis patients [113]. In summary, much of the data supporting the link between RAS-cytokine gene polymorphisms and the progression of renal and CV disease stem from adult studies that involve a significantly larger prevalent population. However, there are preliminary data supporting efforts for genotype–phenotype association studies in children [55–57]. Although there is heterogeneity for the causes of CKD between the adult and pediatric populations, progression for renal and CV abnormalities shares the same final common pathway, as discussed above.

## Finding the link between genotype and phenotype

As stated previously, the natural variations of the genes involving the RAS–cytokine pathway potentially influence the rate of progressions for renal and CV disease in CKD patients. For example, a study by Balakrishnan et al. demonstrates the relationship between cytokine gene polymorphism and cytokine secretion from peripheral blood monocytes (PBMC) in hemodialysis patients [47]. Genotyping was performed for SNPs in the promoter region of IL-6 (-174 G>C), TNF-α (-308G>A), and IL-10 (-1082 G>A) in 183 ESRD patients. Plasma cytokine levels by endotoxin-stimulated PBMCs were measured by enzyme-linked immunosorbent assay (ELISA). Plasma IL-6 levels were higher in the circulating blood from patients having -174G/C or C/C genotype; and IL-10 secretion was increased in -1082 G/G genotype (Table 3). The inflammatory response to the uremic milieu is variable and associated with cytokine gene polymorphisms in CKD patients***.***

**Table 3:** Relationship between genotype and plasma cytokine levels [72]

Cytokine	Genotype	Transcription/secretion level (expected)	Plasma level in pg/ml mean ± SD
Interleukin (IL)-6	-174 C/C	Low	12.2 ± 5.1
	-174 G/G, G/C	High	15.01 ± 17.4^a^
Tumor necrosis factor (TNF)-α	-308 G/G	Low	998.8 ± 1156.2
	-308 G/A, A/A	High	1131 ± 1616.2
IL-10	-1082 A/A	Low	344.8 ± 356.3
	-1082 G/A	Intermediate	391.0 ± 440.5
	-1082 G/G	High	627.4 ± 506.2^b^

^a^Levene’s test for unequal variance,* p*=0.05, ^b^Kruskall–Wallis test,* p* = 0.01

By analyzing differences in DNA sequences in large cohort studies, the disease phenotype can be mapped according to known genetic markers (i.e. known locations of SNPs) by linkage analysis. Unlike monogenic disorders, polygenic disorders as in CKD and CV abnormalities are the result of complex interactions of intra- and intercellular systems that are governed by multiple gene loci (polygenic), often modified by gene–environment and gene–gene interactions (epistasis) [24]. The genetic determinants of renal and CV disease progression are cumulative variations of gene transcription and function in these interdependent pathways.

A consistent picture of genotype–phenotype relationships in CKD and CV disease are lacking, probably because: (1) other unknown functional loci may be present; (2) polymorphisms that are known but which have not yet been recognized to be functional may exist; (3) inconsistent definitions of CKD; (4) heterogeneity of diseases that cause CKD; (5) limitations due to sample size, especially in the pediatric CKD population. Polymorphisms do not exist in isolation, and it may be the combination of base changes at several proximal sites along the allele, i.e. the haplotype that influences the function. Haplotype methods may capture a large proportion of the genetic variation across sizable regions using a minimal number of tag SNPs [114, 115], as discussed further below.

## Haplotype and HapMap

Alleles that are in close proximity along a DNA strand tend to cross over together during recombination and comprise a haplotype. In a population, common haplotypes can be inherited among many individuals from a common ancestor, with complementary haplotypes being given by each parent. The characterization haplotype variants among human populations (https://doi.org/www.hapmap.org) offers new opportunities in the genetic analysis of CKD through whole-genome association studies.

At this time, the International Haplotype Map (HapMap) Consortium is characterizing where and how frequently sequence variants occur in the human genome in four different ethnic groups worldwide [116]. Here, SNPs along a chromosome that tend to be inherited together can define a haplotype. Although a chromosomal region may contain many SNPs, there only a few tag SNPs that offer the most information about genetic variation in that region. Within candidate genes, the number of common polymorphisms is finite [117], but direct assay of all existing common polymorphism is inefficient, because genotypes at many of these sites are strongly correlated. Selection of the maximally informative set of a common tag SNP set can comprehensively interrogate for main effects of the haplotype [118, 119].

The HapMap project has opened the way for whole-genome association studies. Association refers to the statistical dependence between two variables, which is a measure of the degree to which the frequency of a risk factor, in this case a genetic marker, is different between persons with disease compared with those without disease. Hence, studies to determine the genes that influence CKD progression will compare CKD patients with a more rapid decline in glomerular filtration rate (GFR) to those with slower progression. Regions where the two groups differ in their haplotype frequencies might contain genes associated with renal progression. However, the dawning of new technology for rapid sequencing of DNA has implications of advancing this field of research beyond the HapMap [120].

## Potential limitations of gene association studies

The presence of an association does not imply that the observed relationship is one of cause and effect. A judgment of causation from epidemiologic data relies on assessing the validity of the observed statistical association and the cumulative evidence from a variety of sources in order to support a causal inference regarding the genetic marker of interest. An association between a gene and disease may be indicative of a true relationship between the gene and the disease; however, the association may not be valid due to chance (type I error), limited sample size (type II error), bias, or confounding. Ideally, a gene association study will have sufficient cases and controls (on the order of thousands) to identify a gene locus or region that is truly associated with the disease given a threshold of significance on the order of *P* value < 10^−6^ [121]. However, an investigation of this nature is not likely to be achieved immediately in the pediatric CKD population without preliminary evidence from studies of modest sizes varying in degrees of quality. Furthermore, no single center alone will have a sufficient number of pediatric CKD subjects to power such an analysis and would likely need a cooperative effort among a number of pediatric CKD centers.

### Type I and type II error

Typically, investigators may submit their data sets for genotyping with a standard, very large, set of SNPs, which will include markers located in the candidate gene along with a few thousand which are not. Spurious associations where the genetic marker of interest is correlated with the disease by chance may occur, especially in the presence of multiple comparisons [122]. To illustrate the multiple comparisons problem, we use a hypothetical case-control study of 10,000 unrelated SNPs to be tested for association with the disease of interest, with the threshold for statistical significance set to a* P* value of 0.01. If 10,000 statistical tests are performed to assess the association between each of the SNPs and the disease, by chance alone, we would expect 100 SNPs at random to be statistically associated with the disease, even though there is no true relationship; this is a type I error. With increasing number of genetic markers being typed and multiple intermediate phenotypes being tested, strict guidelines for publication of gene association studies have been proposed [123–125].

In studies of modest size, SNP markers in the candidate gene may produce relatively modest evidence in favor of association with a level of* P* < 0.01, even with hundreds of subjects [126]. Thus, a marker having a real but modest effect is not expected to produce an odds ratio (OR) with a smaller* P* value than markers producing apparently significant results by chance [126]. In addressing this limitation, setting a stringent* P* value (*P* < 10^−6^) is a generally accepted convention for large studies with markers of low prior probability of true association [121]. Applicable to studies of smaller magnitude, alternative methods relying on Bayesian strategies have been proposed [126, 127]. Under these approaches, the prior probability of association based on preliminary evidence is used to weight the* P* value obtained. This supports the general recommendation for having biologic plausibility of the observed association such that the genetic variant of interest is involved the pathogenesis of disease [122, 124, 128]. It is worth considering whether the known function of the gene is linked to the observed phenotype. Furthermore, the association between gene and disease is less likely to be spurious if the relationship is observed in other independent studies. Although mildly controversial [129], there is general agreement that the initial findings of a gene–disease association study be replicated by other studies [130, 131].

Many gene association studies are not replicated for a number of reasons. A small sample size may limit a study’s ability to detect an association if one truly exists. Concluding that there is no association when one truly exists defines a type II error. The magnitude of the contribution of single gene variants to polygenic disorders is small, with a typical effect sizes corresponding to ORs of 1.2–1.6 [132]. Compared with the smaller sample sizes needed to detect a larger effect (e.g. OR ≥ 2.0), detecting associations of smaller magnitude requires a much larger sample size, which may dramatically increase the recruitment costs and make some studies unfeasible [132].

Furthermore, the lack of consistency in proposed gene–disease association across studies may also be reflective in the inherent complexity and heterogeneity of common diseases, including CKD [131], which are beyond the scope of this review. A proposed solution for studies with insufficient power are to: (1) emphasize replication and obtain data to determine biologic plausibility; (2) synthesize results of individual studies for meta-analysis; or (3) obtain data on individual subjects from several studies to perform a pooled analysis [125].

### Limitations in study design

Biased estimates for an observed association between genotype and phenotype may originate from a flaw in the design or conduct of the study that has introduced systematic error or bias into the result. There are numerous considerations to this point, but a few topics deserve some mention in this general overview. A more complete discussion of these considerations can be found elsewhere [122, 125, 133, 134]. The adequacy of any epidemiologic study design depends on the scientific question [135]. A study that is designed to detect an association may “overselect” cases in order to detect an association. However, the measures of the association from studies designed to detect genotype–phenotype associations are not valid or generalizable measures of association for the population [125].

The case–control study is a common study paradigm for genome association studies because it is economically efficient, allows for the evaluation of diseases with long latent periods, and can examine multiple etiologic factors for one disease; however compared with other study paradigms, case–control studies are particularly prone to bias if controls are not properly selected [136]. Genetic association studies may be biased or confounded by population stratification [133] and genotyping errors [134]. Population stratification can create the appearance of a SNP–disease association and arises when race and/or ethnicity is related both to the SNP under investigation (e.g. differences in allele frequencies within distinct ethnic groups) and to the disease of interest. Similar to epidemiologic studies needing to address race and ethnicity as potential confounders, gene polymorphism studies should assess the potential for bias and confounding due to population stratification [137].

Of additional concern, genotyping errors can be significant, leading to null results or erroneous conclusions [134]. Genotyping errors can stem from a number of causes, including: (1) inadequate sample quality; (2) artifacts due to biochemical or equipment problems; (3) errors from the DNA amplification process; (4) human factors. Genotyping errors in phenotype–genotype association studies will tend to bias estimates of association toward the null assuming that errors occur at equal frequency across case status [134]. However, if cases and controls are genotyped using different assays or run separately in distinct batches, differential errors may occur resulting in either over- or underestimation of the true association [134].

## Conclusions

Genetic association studies have the potential to provide new insights into the factors responsible for CKD renal and CV progression. These investigations provide hope for new drug targets to treat or modify individual disease risk. In the case of CKD, genetic polymorphisms in the RAS–cytokine pathway may be responsible for the intraindividual variation in renal and cardiac progression in patients with CKD and may offer new targets for drug therapy.

The Human Genome and HapMap projects have made it possible to evaluate a multitude of candidate genes that might be linked to CKD progression. The enthusiasm for these investigations must be tempered by acknowledging the limitations of gene association studies. Attention to biologic plausibility and appropriate study design will help the interpretability of published results. Independent investigations replicating initial findings are needed to support an inference of a causal association between the gene polymorphism of interest and the disease phenotype.

Whole-genome association studies are becoming widely available and are being performed to investigate the genetic predisposition to diabetic nephropathy [49, 50]. In an effort to understand the risk factors for progression of CKD and CV disease, the ongoing CKD cohort studies in adult and pediatric patients [138, 139] are evaluating known risk factors for CKD progression, including etiology of CKD, proteinuria, and hypertension. These cohort studies are collecting biologic and genetic samples for future studies of cytokines or their genetic polymorphisms, which may yield scientific insight into the pathophysiologic mechanisms of CKD progression in both adults and children.

## Questions

(Answers appear following the reference list)

1. Which of the statements below is **not** a characteristic of angiotensin II (AT2)?
  A AT2 is important for regulating blood pressure.
  B AT2 is produced locally in the vascular beds of the kidney and the heart.
  C AT2 is involved in myocardial hypertrophy.
  D AT2 is not involved in cytokine signaling.
  E AT2 is a factor regulating progressive kidney injury.
2. Where do functional single nucleotide polymorphisms (SNPs) occur?
  A Gene promoter regions.
  B Splice junctions.
  C 3′-untranslated regions.
  D All the above.
  E A and C only.
3. A consistent picture of genotype–phenotype relationships in CKD and CV disease are lacking because:
  A Other unknown functional loci may be present.
  B The studies investigating such relationships have been performed in one ethnic population.
  C There are known polymorphisms but they are not recognized to be functional.
  D All the above.
  E A and C only.
4. A reported genotype–phenotype association may not be valid due to the following **except**:
  A Type II error
  B Type III error
  C There is no biologic plausibility for the finding.
  D There might be a spurious statistical association due to chance.
  E Investigators did not account for population stratification.
5. For a hypothetical case–control study to evaluate gene cytokine polymorphisms in patients with chronic kidney disease, genotyping errors in a may occur in the following circumstances:
  A Whereas patients with CKD have DNA extracted from white blood cells, the control population has DNA extracted from buccal cells.
  B DNA amplification for cases and controls are analyzed in the same lab, by the same amplification process, and by the same technician.
  C The technician performing the DNA amplification is inexperienced and has little supervision from the principal investigator.
  D All the above.
  E A and C only.

Answers:

1. D
2. D
3. E
4. B
5. E

## References

[1] Wong CS, Furth SL (2007) Epidemiology of renal disease in children. In: Kher KK, Schnaper HW, Makker SP (eds) Clinical Pediatric Nephrology. Informa UK Ltd, London, pp 63–70

[2] Wong CS, Mak RH, Kher KK, Schnaper HW, Makker SP (2007). Chronic kidney disease. Clinical pediatric nephrology.

[3] United States Renal Data System (2007) USRDS 2007 Annual Data Report: Atlas of chronic kidney disease and end stage renal disease in the United States. In: the National Institutes of Health, National Institute of Diabetes and Digestive and Kidney Diseases. Bethesda, MD

[4] NAPRTCS (2007) 2007 Annual Report. EMMES, Rockville, MD

[5] Ardissino G, Dacco V, Testa S, Bonaudo R, Claris-Appiani A, Taioli E, Marra G, Edefonti A, Sereni F, ItalKid Project (2003). Epidemiology of chronic renal failure in children: data from the ItalKid project. Pediatrics.

[6] Soares CM, Oliveira EA, Diniz JS, Lima EM, Vasconcelos MM, Oliveira GR (2003). Predictive factors of progression of chronic renal insufficiency: a multivariate analysis. Pediatr Nephrol.

[7] Mitsnefes M, Ho PL, McEnery PT (2003). Hypertension and progression of chronic renal insufficiency in children: a report of the North American Pediatric Renal Transplant Cooperative Study (NAPRTCS). J Am Soc Nephrol.

[8] Wingen AM, Fabian-Bach C, Schaefer F, Mehls O (1997). Randomised multicentre study of a low-protein diet on the progression of chronic renal failure in children. European Study Group of nutritional treatment of chronic renal failure in childhood. Lancet.

[9] Seikaly MG, Ho PL, Emmett L, Fine RN, Tejani A (2003). Chronic renal insufficiency in children: The 2001 Annual Report of the NAPRTCS. Pediatr Nephrol.

[10] Eddy AA (2005). Progression in chronic kidney disease. Adv Chronic Kidney Dis.

[11] Ruiz-Ortega M, Lorenzo O, Suzuki Y, Ruperez M, Egido J (2001). Proinflammatory actions of angiotensins. Curr Opin Nephrol Hypertens.

[12] Go AS, Chertow GM, Fan D, McCulloch CE, Hsu CY (2004). Chronic kidney disease and the risks of death, cardiovascular events, and hospitalization. N Engl J Med.

[13] Sarnak MJ, Levey AS, Schoolwerth AC, Coresh J, Culleton B, Hamm LL, McCullough PA, Kasiske BL, Kelepouris E, Klag MJ, Parfrey P, Pfeffer M, Raij L, Spinosa DJ, Wilson PW (2003). Kidney disease as a risk factor for development of cardiovascular disease: a statement from the American Heart Association Councils on kidney in cardiovascular disease, high blood pressure research, clinical cardiology, and epidemiology and prevention. Hypertension.

[14] Meyer KB, Levey AS (1998). Controlling the epidemic of cardiovascular disease in chronic renal disease; report from the National Kidney Foundation Task Force on cardiovascular disease. J Am Soc Nephrol.

[15] Parekh RS, Carroll CE, Wolfe RA, Port FK (2002). Cardiovascular mortality in children and young adults with end-stage kidney disease. J Pediatr.

[16] Chavers BM, Li S, Collins AJ, Herzog CA (2002). Cardiovascular disease in pediatric chronic dialysis patients. Kidney Int.

[17] Merouani A, Lambert M, Delvin EE, Genest J, Robitaille P, Rozen R (2001). Plasma homocysteine concentration in children with chronic renal failure. Pediatr Nephrol.

[18] Siirtola A, Antikainen M, Ala-Houhala M, Koivisto AM, Solakivi T, Jokela H, Lehtimaki T, Holmberg C, Salo MK (2004). Serum lipids in children 3 to 5 years after kidney, liver, and heart transplantation. Transpl Int.

[19] Saland JM, Ginsberg H, Fisher EA (2002). Dyslipidemia in pediatric renal disease: epidemiology, pathophysiology, and management. Curr Opin Pediatr.

[20] Mitsnefes MM, Barletta GM, Dresner IG, Chand DH, Geary D, Lin JJ, Patel H (2006). Severe cardiac hypertrophy and long-term dialysis: the Midwest Pediatric Nephrology Consortium study. Pediatr Nephrol.

[21] Mitsnefes MM, Kimball TR, Witt SA, Glascock BJ, Khoury PR, Daniels SR (2003). Left ventricular mass and systolic performance in pediatric patients with chronic renal failure. Circulation.

[22] Mitsnefes MM (2002). Pediatric end-stage renal disease: heart as a target. J Pediatr.

[23] Tsagalis G, Zerefos S, Zerefos N (2007). Cardiorenal syndrome at different stages of chronic kidney disease. Int J Artif Organs.

[24] Rao M, Wong C, Kanetsky P, Girndt M, Stenvinkel P, Reilly M, Raj DS (2007). Cytokine gene polymorphism and progression of renal and cardiovascular diseases. Kidney Int.

[25] Ruiz-Ortega M, Esteban V, Ruperez M, Sanchez-Lopez E, Rodriguez-Vita J, Carvajal G, Egido J (2006). Renal and vascular hypertension-induced inflammation: role of angiotensin II. Curr Opin Nephrol Hypertens.

[26] The GISEN Group (Gruppo Italiano di Studi Epidemiologici in Nefrologia) (1997). Randomised placebo-controlled trial of effect of ramipril on decline in glomerular filtration rate and risk of terminal renal failure in proteinuric, non-diabetic nephropathy. The GISEN Group (Gruppo Italiano di Studi Epidemiologici in Nefrologia). Lancet.

[27] Maschio G, Alberti D, Janin G, Locatelli F, Mann JF, Motolese M, Ponticelli C, Ritz E, Zucchelli P (1996). Effect of the angiotensin-converting-enzyme inhibitor benazepril on the progression of chronic renal insufficiency. The Angiotensin-Converting-Enzyme Inhibition in Progressive Renal Insufficiency Study Group. N Engl J Med.

[28] Jafar TH, Stark PC, Schmid CH, Landa M, Maschio G, de Jong PE, de Zeeuw D, Shahinfar S, Toto R, Levey AS (2003). Progression of chronic kidney disease: the role of blood pressure control, proteinuria, and angiotensin-converting enzyme inhibition: a patient-level meta-analysis. Ann Intern Med.

[29] Jacoby DS, Rader DJ (2003). Renin-angiotensin system and atherothrombotic disease: from genes to treatment. Arch Intern Med.

[30] Ruiz-Ortega M, Ruperez M, Lorenzo O, Esteban V, Blanco J, Mezzano S, Egido J (2002) Angiotensin II regulates the synthesis of proinflammatory cytokines and chemokines in the kidney. Kidney Int Suppl (82):12–22 10.1046/j.1523-1755.62.s82.4.x 12410849

[31] Esteban V, Lorenzo O, Ruperez M, Suzuki Y, Mezzano S, Blanco J, Kretzler M, Sugaya T, Egido J, Ruiz-Ortega M (2004). Angiotensin II, via AT1 and AT2 receptors and NF-kappa B pathway, regulates the inflammatory response in unilateral ureteral obstruction. J Am Soc Nephrol.

[32] Brasier AR, Jamaluddin M, Han Y, Patterson C, Runge MS (2000). Angiotensin II induces gene transcription through cell-type-dependent effects on the nuclear factor-kappa B (NF-kappa B) transcription factor. Mol Cell Biochem.

[33] Ruiz-Ortega M, Lorenzo O, Ruperez M, Blanco J, Egido J (2001). Systemic infusion of angiotensin II into normal rats activates nuclear factor-kappa B and AP-1 in the kidney: role of AT(1) and AT(2) receptors. Am J Pathol.

[34] Raj DS, Shah H, Shah VO, Ferrando A, Bankhurst A, Wolfe R, Zager PG (2003). Markers of inflammation, proteolysis, and apoptosis in ESRD. Am J Kidney Dis.

[35] Klahr S, Morrissey J (2003). Progression of chronic renal disease. Am J Kidney Dis.

[36] Hogg RJ, Portman RJ, Milliner D, Lemley KV, Eddy A, Ingelfinger J (2000). Evaluation and management of proteinuria and nephrotic syndrome in children: recommendations from a pediatric nephrology panel established at the national kidney foundation conference of proteinuria, albuminuria, risk, assessment, detection, and elimination (PARADE). Pediatrics.

[37] Drumm K, Bauer B, Freudinger R, Gekle M (2002). Albumin induces NF-kappa B expression in human proximal tubule-derived cells (IHKE-1). Cell Physiol Biochem.

[38] Zoja C, Benigni A, Remuzzi G (2004). Cellular responses to protein overload: key event in renal disease progression. Curr Opin Nephrol Hypertens.

[39] Takase O, Marumo T, Imai N, Hirahashi J, Takayanagi A, Hishikawa K, Hayashi M, Shimizu N, Fujita T, Saruta T (2005). NF-kappa B-dependent increase in intrarenal angiotensin II induced by proteinuria. Kidney Int.

[40] Tarone G, Lembo G (2003). Molecular interplay between mechanical and humoral signalling in cardiac hypertrophy. Trends Mol Med.

[41] Lijnen P, Petrov V (1999). Antagonism of the renin-angiotensin system, hypertrophy and gene expression in cardiac myocytes. Methods Find Exp Clin Pharmacol.

[42] Dostal DE, Hunt RA, Kule CE, Dostal DE, Hunt RA, Kule CE, Bhat GJ, Karoor V, McWhinney CD, Baker KM (1997). Molecular mechanisms of angiotensin II in modulating cardiac function: intracardiac effects and signal transduction pathways. J Mol Cell Cardiol.

[43] Lijnen P, Petrov V (1999). Renin-angiotensin system, hypertrophy and gene expression in cardiac myocytes. J Mol Cell Cardiol.

[44] Murray DR, Prabhu SD, Chandrasekar B (2000). Chronic beta-adrenergic stimulation induces myocardial proinflammatory cytokine expression. Circulation.

[45] Petrov VV, Fagard RH, Lijnen PJ (2000). Transforming growth factor-beta(1) induces angiotensin-converting enzyme synthesis in rat cardiac fibroblasts during their differentiation to myofibroblasts. J Renin Angiotensin Aldosterone Syst.

[46] Park J-Y, Farrance IKG, Fenty NM, Hagberg JM, Roth SM, Mosser DM, Wang MQ, Jo H, Okazaki T, Brant SR, Brown MD (2007). NFKB1 promoter variation implicates shear-induced NOS3 gene expression and endothelial function in prehypertensives and stage I hypertensives. Am J Physiol Heart Circ Physiol.

[47] Balakrishnan VS, Guo D, Rao M, Jaber BL, Tighiouart H, Freeman RL, Huang C, King AJ, Pereira BJ (2004). Cytokine gene polymorphisms in hemodialysis patients: association with comorbidity, functionality, and serum albumin. Kidney Int.

[48] Rao M, Jaber BL, Balakrishnan VS (2005). Gene polymorphism association studies in dialysis: cardiovascular disease. Semin Dial.

[49] Hadjadj S, Tarnow L, Forsblom C, Kazeem G, Marre M, Groop P-H, Parving H-H, Cambien F, Tregouet DA, Gut IG, Theva A, Gauguier D, Farrall M, Cox R, Matsuda F, Lathrop M, Hager-Vionnet N, EURAGEDIC (European Rational Approach for Genetics of Diabetic Complications) Study Group (2007). Association between angiotensin-converting enzyme gene polymorphisms and diabetic nephropathy: case-control, haplotype, and family-based study in three European populations. J Am Soc Nephrol.

[50] Iyengar SK, Abboud HE, Goddard KA, Saad MF, Adler SG, Arar NH, Bowden DW, Duggirala R, Elston RC, Hanson RL, Ipp E, Kao WH, Kimmel PL, Klag MJ, Knowler WC, Meoni LA, Nelson RG, Nicholas SB, Pahl MV, Parekh RS, Quade SR, Rich SS, Rotter JI, Scavini M, Schelling JR, Sedor JR, Sehgal AR, Shah VO, Smith MW, Taylor KD, Winkler CA, Zager PG, Freedman BI (2007). Genome-wide scans for diabetic nephropathy and albuminuria in multiethnic populations: the family investigation of nephropathy and diabetes (FIND). Diabetes.

[51] Kim JA, Berliner JA, Nadler JL (1996). Angiotensin II increases monocyte binding to endothelial cells. Biochem Biophys Res Commun.

[52] Krejcy K, Eichler HG, Jilma B, Kapiotis S, Wolzt M, Zanaschka G, Gasic S, Schutz W, Wagner O (1996). Influence of angiotensin II on circulating adhesion molecules and blood leukocyte count in vivo. Can J Physiol Pharmacol.

[53] Boright AP, Paterson AD, Mirea L, Bull SB, Mowjoodi A, Scherer SW, Zinman B (2005). Genetic variation at the ACE gene is associated with persistent microalbuminuria and severe nephropathy in type 1 diabetes: the DCCT/EDIC Genetics Study. Diabetes.

[54] Ng DP, Placha G, Choo S, Chia KS, Warram JH, Krolewski AS (2006). A disease haplotype for advanced nephropathy in type 2 diabetes at the ACE locus. Diabetes.

[55] Hohenfellner K, Wingen AM, Nauroth O, Wuhl E, Mehls O, Schaefer F (2001). Impact of ACE I/D gene polymorphism on congenital renal malformations. Pediatr Nephrol.

[56] Papp F, Friedman AL, Bereczki C, Haszon I, Kiss E, Endreffy E, Turi S (2003). Renin-angiotensin gene polymorphism in children with uremia and essential hypertension. Pediatr Nephrol.

[57] Haszon I, Friedman AL, Papp F, Bereczki C, Baji S, Bodrogi T, Karoly E, Endreffy E, Turi S (2002). ACE gene polymorphism and renal scarring in primary vesicoureteric reflux. Pediatr Nephrol.

[58] Lovati E, Richard A, Frey BM, Frey FJ, Ferrari P (2001). Genetic polymorphisms of the renin-angiotensin-aldosterone system in end-stage renal disease. Kidney Int.

[59] Raizada V, Skipper B, Luo W, Garza L, Hines CW, Harford AA, Zager PG, Griffith J, Raj D, Spalding CT (2005). Renin-angiotensin polymorphisms and QTc interval prolongation in end-stage renal disease. Kidney Int.

[60] Osono E, Kurihara S, Hayama N, Sakurai Y, Ohwada K, Onoda N, Takeuchi M, Tomizawa T, Komaba Y, Hashimoto K, Matsunobu S, Yoneshima H, Iino Y (1998). Insertion/deletion polymorphism in intron 16 of the ACE gene and left ventricular hypertrophy in patients with end-stage renal disease. Am J Kidney Dis.

[61] Caulfield M, Lavender P, Farrall M, Munroe P, Lawson M, Turner P, Clark AJ (1994). Linkage of the angiotensinogen gene to essential hypertension. N Engl J Med.

[62] Baudin B (2005). Polymorphism in angiotensin II receptor genes and hypertension. Exp Physiol.

[63] Renner W, Nauck M, Winkelmann BR, Hoffmann MM, Scharnagl H, Mayer V, Boehm BO, Marz W (2005). Association of angiotensinogen haplotypes with angiotensinogen levels but not with blood pressure or coronary artery disease: the Ludwigshafen Risk and Cardiovascular Health Study. J Mol Med.

[64] Hsu CC, Bray MS, Kao WH, Pankow JS, Boerwinkle E, Coresh J (2006). Genetic variation of the renin-angiotensin system and chronic kidney disease progression in black individuals in the atherosclerosis risk in communities study. J Am Soc Nephrol.

[65] Curnow KM, Pascoe L, White PC (1992). Genetic analysis of the human type-1 angiotensin II receptor. Mol Endocrinol.

[66] Buraczynska M, Ksiazek P, Drop A, Zaluska W, Spasiewicz D, Ksiazek A (2006). Genetic polymorphisms of the renin-angiotensin system in end-stage renal disease. Nephrol Dial Transplant.

[67] Bonnardeaux A, Davies E, Jeunemaitre X, Fery I, Charru A, Clauser E, Tiret L, Cambien F, Corvol P, Soubrier F (1994). Angiotensin II type 1 receptor gene polymorphisms in human essential hypertension. Hypertension.

[68] Tiret L, Bonnardeaux A, Poirier O, Ricard S, Marques-Vidal P, Evans A, Arveiler D, Luc G, Kee F, Ducimetiere P, Soubrier F, Camblen F (1994). Synergistic effects of angiotensin-converting enzyme and angiotensin-II type 1 receptor gene polymorphisms on risk of myocardial infarction. Lancet.

[69] Arend WP (2002). The balance between IL-1 and IL-1Ra in disease. Cytokine Growth Factor Rev.

[70] Arend WP, Malyak M, Guthridge CJ, Gabay C (1998). Interleukin-1 receptor antagonist: role in biology. Annu Rev Immunol.

[71] Biasucci LM, Liuzzo G, Fantuzzi G, Caligiuri G, Rebuzzi AG, Ginnetti F, Dinarello CA, Maseri A (1999). Increasing levels of interleukin (IL)-1Ra and IL-6 during the first 2 days of hospitalization in unstable angina are associated with increased risk of in-hospital coronary events. Circulation.

[72] Balakrishnan VS, Jaber BL, Natov SN, Cendoroglo M, King AJ, Schmid CH, Pereira BJ (1998). Interleukin-1 receptor antagonist synthesis by peripheral blood mononuclear cells in hemodialysis patients. Kidney Int.

[73] Wetmore JB, Hung AM, Lovett DH, Sen S, Quershy O, Johansen KL (2005). Interleukin-1 gene cluster polymorphisms predict risk of ESRD. Kidney Int.

[74] Hurme M, Santtila S (1998). IL-1 receptor antagonist (IL-1Ra) plasma levels are co-ordinately regulated by both IL-1Ra and IL-1beta genes. Eur J Immunol.

[75] Tarlow JK, Blakemore AI, Lennard A, Solari R, Hughes HN, Steinkasserer A, Duff GW (1993). Polymorphism in human IL-1 receptor antagonist gene intron 2 is caused by variable numbers of an 86-bp tandem repeat. Hum Genet.

[76] Pociot F, Molvig J, Wogensen L, Worsaae H, Nerup J (1992). A TaqI polymorphism in the human interleukin-1 beta (IL-1 beta) gene correlates with IL-1 beta secretion in vitro. Eur J Clin Invest.

[77] Huang G, Niu T, Peng S, Ling D, Liu J, Zhang X, Xu X (2004). Association between the interleukin-1beta C(-511)T polymorphism and blood pressure in a Chinese hypertensive population. Immunol Lett.

[78] Dewberry RM, Crossman DC, Francis SE (2003). Interleukin-1 receptor antagonist (IL-1RN) genotype modulates the replicative capacity of human endothelial cells. Circ Res.

[79] Francis SE, Camp NJ, Dewberry RM, Gunn J, Syrris P, Carter ND, Jeffery S, Kaski JC, Cumberland DC, Duff GW, Crossman DC (1999). Interleukin-1 receptor antagonist gene polymorphism and coronary artery disease. Circulation.

[80] Ray KK, Camp NJ, Bennett CE, Francis SE, Crossman DC (2002). Genetic variation at the interleukin-1 locus is a determinant of changes in soluble endothelial factors in patients with acute coronary syndromes. Clin Sci (Lond).

[81] Watanabe M, Iwano M, Akai Y, Kurioka H, Nishitani Y, Harada K, Hamano K, Shiiki H (2002). Association of interleukin-1 receptor antagonist gene polymorphism with IgA nephropathy. Nephron.

[82] Amoli MM, Calvino MC, Garcia-Porrua C, Llorca J, Ollier WE, Gonzalez-Gay MA (2004). Interleukin 1beta gene polymorphism association with severe renal manifestations and renal sequelae in Henoch-Schonlein purpura. J Rheumatol.

[83] Buraczynska M, Ksiazek P, Kubit P, Zaluska W (2006). Interleukin-1 receptor antagonist gene polymorphism affects the progression of chronic renal failure. Cytokine.

[84] Chiappelli M, Tampieri C, Tumini E, Porcellini E, Caldarera CM, Nanni S, Branzi A, Lio D, Caruso M, Hoffmann E, Caruso C, Licastro F (2005). Interleukin-6 gene polymorphism is an age-dependent risk factor for myocardial infarction in men. Int J Immunogenet.

[85] Sie MP, Sayed-Tabatabaei FA, Oei HH, Uitterlinden AG, Pols HA, Hofman A, van Duijn CM, Witteman JC (2006). Interleukin 6 -174 g/c promoter polymorphism and risk of coronary heart disease: results from the rotterdam study and a meta-analysis. Arterioscler Thromb Vasc Biol.

[86] Losito A, Kalidas K, Santoni S, Jeffery S (2003). Association of interleukin-6 -174G/C promoter polymorphism with hypertension and left ventricular hypertrophy in dialysis patients. Kidney Int.

[87] Muller-Steinhardt M, Hartel C, Muller B, Kirchner H, Fricke L (2002). The interleukin-6 -174 promoter polymorphism is associated with long-term kidney allograft survival. Kidney Int.

[88] Girndt Matthias, Ulrich Christof, Kaul Harald, Sester Urban, Sester Martina, Köhler Hans (2003). Uremia-associated immune defect: The IL-10–CRP axis. Kidney International.

[89] Turner DM, Williams DM, Sankaran D, Lazarus M, Sinnott PJ, Hutchinson IV (1997). An investigation of polymorphism in the interleukin-10 gene promoter. Eur J Immunogenet.

[90] Eskdale J, Gallagher G, Verweij CL, Keijsers V, Westendorp RG, Huizinga TW (1998). Interleukin 10 secretion in relation to human IL-10 locus haplotypes. Proc Natl Acad Sci U S A.

[91] Guo G, Morrissey J, McCracken R, Tolley T, Klahr S (1999). Role of TNFR1 and TNFR2 receptors in tubulointerstitial fibrosis of obstructive nephropathy. Am J Physiol.

[92] Ridker PM, Rifai N, Pfeffer M, Sacks F, Lepage S, Braunwald E (2000). Elevation of tumor necrosis factor-alpha and increased risk of recurrent coronary events after myocardial infarction. Circulation.

[93] Espinoza M, Aguilera A, Auxiliadora Bajo M, Codoceo R, Caravaca E, Cirugeda A, del Peso G, Hevia C, Selgas R (1999). Tumor necrosis factor alpha as a uremic toxin: correlation with neuropathy, left ventricular hypertrophy, anemia, and hypertriglyceridemia in peritoneal dialysis patients. Adv Perit Dial.

[94] Cesari M, Penninx BW, Newman AB, Kritchevsky SB, Nicklas BJ, Sutton-Tyrrell K, Rubin SM, Ding J, Simonsick EM, Harris TB, Pahor M (2003). Inflammatory markers and onset of cardiovascular events: results from the Health ABC study. Circulation.

[95] Mira JP, Cariou A, Grall F, Delclaux C, Losser MR, Heshmati F, Cheval C, Monchi M, Teboul JL, Riche F, Leleu G, Arbibe L, Mignon A, Delpech M, Dhainaut JF (1999). Association of TNF2, a TNF-alpha promoter polymorphism, with septic shock susceptibility and mortality: a multicenter study. JAMA.

[96] D'Alfonso S, Richiardi PM (1996). An intragenic polymorphism in the human tumor necrosis factor alpha (TNFA) chain-encoding gene. Immunogenetics.

[97] August P, Suthanthiran M (2006). Transforming growth factor beta signaling, vascular remodeling, and hypertension. N Engl J Med.

[98] Mezzano SA, Ruiz-Ortega M, Egido J (2001). Angiotensin II and renal fibrosis. Hypertension.

[99] Zoja C, Corna D, Camozzi D, Cattaneo D, Rottoli D, Batani C, Zanchi C, Abbate M, Remuzzi G (2002). How to fully protect the kidney in a severe model of progressive nephropathy: a multidrug approach. J Am Soc Nephrol.

[100] August Phyllis, Suthanthiran Manikkam (2003). Transforming growth factor beta and progression of renal disease. Kidney International.

[101] Li B, Khanna A, Sharma V, Singh T, Suthanthiran M, August P (1999). TGF-beta 1 DNA polymorphisms, protein levels, and blood pressure. Hypertension.

[102] Schultz Jel J, Witt SA, Glascock BJ, Nieman ML, Reiser PJ, Nix SL, Kimball TR, Doetschman T (2002). TGF-beta 1 mediates the hypertrophic cardiomyocyte growth induced by angiotensin II. J Clin Invest.

[103] Cambien F, Ricard S, Troesch A, Mallet C, Generenaz L, Evans A, Arveiler D, Luc G, Ruidavets JB, Poirier O (1996). Polymorphisms of the transforming growth factor-beta 1 gene in relation to myocardial infarction and blood pressure. The Etude Cas-Temoin de l’Infarctus du Myocarde (ECTIM) Study. Hypertension.

[104] Suthanthiran M, Li B, Song JO, Ding R, Sharma VK, Schwartz JE, August P (2000). Transforming growth factor-beta 1 hyperexpression in African-American hypertensives: a novel mediator of hypertension and/or target organ damage. Proc Natl Acad Sci U S A.

[105] Awad MR, El-Gamel A, Hasleton P, Turner DM, Sinnott PJ, Hutchinson IV (1998). Genotypic variation in the transforming growth factor-beta 1 gene: association with transforming growth factor-beta 1 production, fibrotic lung disease, and graft fibrosis after lung transplantation. Transplantation.

[106] Sato F, Narita I, Goto S, Kondo D, Saito N, Ajiro J, Saga D, Ogawa A, Kadomura M, Akiyama F, Kaneko Y, Ueno M, Sakatsume M, Gejyo F (2004). Transforming growth factor-beta 1 gene polymorphism modifies the histological and clinical manifestations in Japanese patients with IgA nephropathy. Tissue Antigens.

[107] Rao M, Guo D, Jaber BL, Tighiouart H, Pereira BJ, Balakrishnan VS (2004). Transforming growth factor-beta 1 gene polymorphisms and cardiovascular disease in hemodialysis patients. Kidney Int.

[108] Fogo AB (2000). The role of angiotensin II and plasminogen activator inhibitor-1 in progressive glomerulosclerosis. Am J Kidney Dis.

[109] Ma LJ, Nakamura S, Aldigier JC, Rossini M, Yang H, Liang X, Nakamura I, Marcantoni C, Fogo AB (2005). Regression of glomerulosclerosis with high-dose angiotensin inhibition is linked to decreased plasminogen activator inhibitor-1. J Am Soc Nephrol.

[110] Francis CW (2002). Plasminogen activator inhibitor-1 levels and polymorphisms. Arch Pathol Lab Med.

[111] Reis K, Arinsoy T, Derici U, Gonen S, Bicik Z, Soylemezoglu O, Yasavul U, Hasanoglu E, Sindel S (2005). Angiotensinogen and plasminogen activator inhibitor-1 gene polymorphism in relation to chronic allograft dysfunction. Clin Transplant.

[112] Wang AY, Poon P, Lai FM, Yu L, Choi PC, Lui SF, Li PK (2001). Plasminogen activator inhibitor-1 gene polymorphism 4G/4G genotype and lupus nephritis in Chinese patients. Kidney Int.

[113] Aucella F, Margaglione M, Vigilante M, Gatta G, Grandone E, Forcella M, Ktena M, De Min A, Salatino G, Procaccini DA, Stallone C (2003). PAI-1 4G/5G and ACE I/D gene polymorphisms and the occurrence of myocardial infarction in patients on intermittent dialysis. Nephrol Dial Transplant.

[114] Daly MJ, Rioux JD, Schaffner SF, Hudson TJ, Lander ES (2001). High-resolution haplotype structure in the human genome. Nat Genet.

[115] Patil N, Berno AJ, Hinds DA, Barrett WA, Doshi JM, Hacker CR, Kautzer CR, Lee DH, Marjoribanks C, McDonough DP, Nguyen BT, Norris MC, Sheehan JB, Shen N, Stern D, Stokowski RP, Thomas DJ, Trulson MO, Vyas KR, Frazer KA, Fodor SP, Cox DR (2001). Blocks of limited haplotype diversity revealed by high-resolution scanning of human chromosome 21. Science.

[116] The International HapMap Consortium (2005). A haplotype map of the human genome. Nature.

[117] Kruglyak L, Nickerson DA (2001). Variation is the spice of life. Nat Genet.

[118] Johnson GC, Esposito L, Barratt BJ, Smith AN, Heward J, Di Genova G, Ueda H, Cordell HJ, Eaves IA, Dudbridge F, Twells RC, Payne F, Hughes W, Nutland S, Stevens H, Carr P, Tuomilehto-Wolf E, Tuomilehto J, Gough SC, Clayton DG, Todd JA (2001). Haplotype tagging for the identification of common disease genes. Nat Genet.

[119] Carlson CS, Eberle MA, Rieder MJ, Yi Q, Kruglyak L, Nickerson DA (2004). Selecting a maximally informative set of single-nucleotide polymorphisms for association analyses using linkage disequilibrium. Am J Hum Genet.

[120] Check E (2007). Time runs short for HapMap. Nature.

[121] Wang WY, Barratt BJ, Clayton DG, Todd JA (2005). Genome-wide association studies: theoretical and practical concerns. Nat Rev Genet.

[122] Healy DG (2006). Case-control studies in the genomic era: a clinician’s guide. Lancet Neurol.

[123] Freimer NB, Sabatti C (2005). Guidelines for association studies in human molecular genetics. Hum Mol Genet.

[124] Rebbeck TR, Martinez ME, Sellers TA, Shields PG, Wild CP, Potter JD (2004). Genetic variation and cancer: improving the environment for publication of association studies. Cancer Epidemiol Biomarkers Prev.

[125] Little J, Bradley L, Bray MS, Clyne M, Dorman J, Ellsworth DL, Hanson J, Khoury M, Lau J, O’Brien TR, Rothman N, Stroup D, Taioli E, Thomas D, Vainio H, Wacholder S, Weinberg C (2002). Reporting, appraising, and integrating data on genotype prevalence and gene-disease associations. Am J Epidemiol.

[126] Curtis D, Vine AE, Knight J (2007). A pragmatic suggestion for dealing with results for candidate genes obtained from genome wide association studies. BMC Genet.

[127] Pe’er I, de Bakker PI, Maller J, Yelensky R, Altshuler D, Daly MJ (2006). Evaluating and improving power in whole-genome association studies using fixed marker sets. Nat Genet.

[128] Rothman KJ, Greenland S (2005). Causation and causal inference in epidemiology. Am J Public Health.

[129] Vieland VJ (2001). The replication requirement. Nat Genet.

[130] Chanock SJ, Manolio T, Boehnke M, Boerwinkle E, Hunter DJ, Thomas G, Hirschhorn JN, Abecasis G, Altshuler D, Bailey-Wilson JE, Brooks LD, Cardon LR, Daly M, Donnelly P, Fraumeni JF, Freimer NB, Gerhard DS, Gunter C, Guttmacher AE, Guyer MS (2007). Replicating genotype–phenotype associations. Nature.

[131] Rebbeck TR, Khoury MJ, Potter JD (2007). Genetic Association Studies of Cancer: where do we go from here. Cancer Epidemiol Biomarkers Prev.

[132] Ioannidis JP, Trikalinos TA, Khoury MJ (2006). Implications of small effect sizes of individual genetic variants on the design and interpretation of genetic association studies of complex diseases. Am J Epidemiol.

[133] Cardon LR, Palmer LJ (2003). Population stratification and spurious allelic association. Lancet.

[134] Pompanon F, Bonin A, Bellemain E, Taberlet P (2005). Genotyping errors: causes, consequences and solutions. Nat Rev Genet.

[135] Terwilliger JD, Weiss KM (2003). Confounding, ascertainment bias, and the blind quest for a genetic “fountain of youth”. Ann Med.

[136] Hennekens C, Buring J (1987). Case-control studies. Epidemiology in medicine.

[137] Wacholder S, Rothman N, Caporaso N (2000). Population Stratification in Epidemiologic Studies of Common Genetic Variants and Cancer: Quantification of Bias. J Natl Cancer Inst.

[138] Furth SL, Cole SR, Moxey-Mims M, Kaskel F, Mak R, Schwartz G, Wong C, Munoz A, Warady BA (2006). Design and Methods of the Chronic Kidney Disease in Children (CKiD) Prospective Cohort Study. Clin J Am Soc Nephrol.

[139] Feldman HI, Appel LJ, Chertow GM, Cifelli D, Cizman B, Daugirdas J, Fink JC, Franklin-Becker ED, Go AS, Hamm LL, He J, Hostetter T, Hsu CY, Jamerson K, Joffe M, Kusek JW, Landis JR, Lash JP, Miller ER, Mohler ER, Muntner P, Ojo AO, Rahman M, Townsend RR, Wright JT (2003). The Chronic Renal Insufficiency Cohort (CRIC) Study: design and methods. J Am Soc Nephrol.

